# Supplementary material for: Integrative small and long RNA omics analysis of human healing and nonhealing wounds discovers cooperating microRNAs as therapeutic targets
Source: eLife. 2022 Aug 12;11:e80322. doi: 10.7554/eLife.80322 (PMC9374442; doi:10.7554/eLife.80322)
Supplement: Supplementary file 2. [file elife-80322-supp2.docx]

**Supplementary file 2. Quality control of rRNA-depleted total RNA sequencing data.**

| **Groups** | **Sample names** | **Total raw**  **reads** | **Total clean reads** | **Clean data rate (%)** | **Input reads** | **Uniquely mapped reads** | **Uniquely mapped**  **rate(%)** |
| --- | --- | --- | --- | --- | --- | --- | --- |
| VU | VU1 | 101622474 | 95087574 | 0.94 | 47543787 | 41697735 | 0.877 |
| VU | VU2 | 101685312 | 92173926 | 0.91 | 46086963 | 35158771 | 0.7629 |
| VU | VU3 | 132592006 | 126190658 | 0.95 | 63095329 | 55771525 | 0.8839 |
| VU | VU4 | 108786868 | 103438880 | 0.95 | 51719440 | 43012289 | 0.8316 |
| VU | VU5 | 108416676 | 101355626 | 0.93 | 50677813 | 41194500 | 0.8129 |
| Skin | Skin1 | 138065658 | 129299094 | 0.94 | 64649547 | 55300913 | 0.8554 |
| Skin | Skin2 | 102996410 | 97056760 | 0.94 | 48528380 | 38950588 | 0.8026 |
| Skin | Skin3 | 123478422 | 116018720 | 0.94 | 58009360 | 47207545 | 0.8138 |
| Skin | Skin4 | 129515874 | 115240764 | 0.89 | 57620382 | 48834112 | 0.8475 |
| Skin | Skin5 | 109239608 | 103341784 | 0.95 | 51670892 | 44434490 | 0.86 |
| Wound1 | Wound1_1 | 105127142 | 100922792 | 0.96 | 50461396 | 44012941 | 0.8722 |
| Wound1 | Wound1_2 | 104542368 | 100678896 | 0.96 | 50339448 | 44040712 | 0.8749 |
| Wound1 | Wound1_3 | 126002796 | 119469842 | 0.95 | 59734921 | 51566913 | 0.8633 |
| Wound1 | Wound1_4 | 103667154 | 98633466 | 0.95 | 49316733 | 41945529 | 0.8505 |
| Wound1 | Wound1_5 | 111446060 | 106610264 | 0.96 | 53305132 | 45076334 | 0.8456 |
| Wound7 | Wound7_1 | 115447748 | 109997276 | 0.95 | 54998638 | 45453970 | 0.8265 |
| Wound7 | Wound7_2 | 104662588 | 100033286 | 0.96 | 50016643 | 42515374 | 0.85 |
| Wound7 | Wound7_3 | 102504766 | 97652674 | 0.95 | 48826337 | 42134005 | 0.8629 |
| Wound7 | Wound7_4 | 108449990 | 103504388 | 0.95 | 51752194 | 43379024 | 0.8382 |
| Wound7 | Wound7_5 | 129466364 | 123563104 | 0.95 | 61781552 | 51511479 | 0.8338 |

Raw reads, clean reads, input reads, and mapped reads are paired fragments.
